# Association of the triglyceride-glucose index with the occurrence and recurrence of colorectal adenomas: a retrospective study from China

**DOI:** 10.1186/s12889-024-18076-x

**Published:** 2024-02-23

**Authors:** Jiaoyan Li, Jingfeng Chen, Haoshuang Liu, Su Yan, Youxiang Wang, Miao Xing, Suying Ding

**Affiliations:** 1 Health Management Center, the First Affiliated Hospital of Zhengzhou University, Longhu Middle Ring Road, Jinshui District, Zhengzhou, 450052 Henan China; 2 College of Public Health, Zhengzhou University, Zhengzhou, 450000 China; 3 Henan Provincial Center for Disease Control and Prevention, Zhengzhou, 450018 China; 4 School of Basic Medicine and Forensic Medicine, Henan University of Science and Technology, Luoyang, China

**Keywords:** Colorectal cancer, Colorectal adenoma, Recurrent adenoma, Insulin resistance, TyG index

## Abstract

**Background:**

Resection of colorectal adenoma (CRA) prevents colorectal cancer; however, recurrence is common. We aimed to assess the association of the triglyceride-glucose (TyG) index with CRA occurrence and recurrence.

**Methods:**

Data from 3392 participants at a hospital in China from 2020 to 2022 were analyzed. Logistic regression was used to estimate odds ratios (ORs) and 95% confidence intervals (CIs). A restricted cubic spline was used to fit TyG index dose‒response curves to recurrent adenomas. The discriminatory power of TyG index for predicting later recurrence was assessed with the area under the receiver operating characteristic (ROC) curve in 170 patients with a TyG index at initial adenoma diagnosis.

**Results:**

One thousand five hundred ninety-six adenoma and 1465 normal participants were included in the occurrence analysis, and 179 recurrent and 152 nonrecurrent participants were included in the recurrence analysis. The TyG mutation was an independent risk factor for CRA occurrence and recurrence. After adjusting for confounders, the risk of adenoma in the participants in Q2, Q3, and Q4 groups of TyG was 1.324 (95% CI 1.020–1.718), 1.349 (95% CI 1.030–1.765), and 1.445 (95% CI 1.055–1.980) times higher than that of the Q1, respectively, and the risk of recurrence in the Q3 and Q4 groups was 2.267 (95% CI 1.096–4.691) and 2.824 (95% CI 1.199–6.648) times in Q1 group. Multiple logistic regression showed that the highest quartile of the TyG index was associated with a greater risk of advanced adenoma recurrence (OR 4.456, 95% CI 1.157–17.164), two or more adenomas (OR 5.079, 95% CI 1.136–22.714 [after removal of TyG index extreme values]), and proximal colon or both adenomas (OR 3.043, 95% CI 1.186–7.810). Subgroup analysis revealed that the association was found to be present only in participants of all age groups who were either male or without obesity, hyperglycemia, hypertension, or dyslipidemia (*p* < 0.05). ROC curves illustrated that the TyG index had good predictive efficacy for identifying recurrence, especially for patients with two or more adenomas (AUC 0.777, 95% CI 0.648–0.907).

**Conclusions:**

An increase in the TyG index is associated with an increased risk of adenoma occurrence and recurrence, with a stronger association with the latter.

**Supplementary Information:**

The online version contains supplementary material available at 10.1186/s12889-024-18076-x.

## Background

Colorectal cancer (CRC) is one of the most common gastrointestinal malignancies, and more than 1.9 million new cases of CRC, accounting for 10% of diagnosed cancers and 935,000 deaths, accounting for 9.4% of the leading causes of cancer death, were estimated to exist in 2020; CRC ranks third and second in global malignancies, respectively [1], and seriously affects people's health. Colorectal adenoma (CRA) are recognized as precancerous lesions of CRC, and colonoscopy screening and timely removal of precancerous lesions could reduce CRC incidence and mortality [2–4]. However, several studies have shown that the risk of CRC may increase after resection of adenomas of higher risk categories compared to that in the general population [5, 6]. The high recurrence rate [7] after resection of adenoma may explain this difference. Several studies have shown that adenomas in higher risk categories are common following polypectomy [8], increasing the risk of CRC. Therefore, adenoma patients still need regular monitoring after resection. However, to reduce pressure during colonoscopy and improve its effectiveness, more convenient and accessible monitoring indicators should be explored, as these indicators are highly valuable for CRC prevention.

Insulin resistance (IR) and hyperinsulinemia play key roles in the pathogenesis of CRC [9–11], as verified by extensive epidemiological data [12, 13], and may even play important roles in the early stages of the adenoma-carcinoma pathway [14, 15]; however, it is unclear whether these conditions lead to adenoma recurrence. The triglyceride-glucose (TyG) index is a parameter derived from fasting blood glucose (FBG) and triglyceride (TG) levels and has been evaluated as a reliable surrogate for IR [16–19]. The TyG index is strongly correlated with the euglycemic-hyperinsulinemic clamp test [20], the gold standard for insulin sensitivity, and has good predictive value for IR (sensitivity of 96.5%, specificity of 85.0%, and area under the curve (AUC) of 0.858), which is comparable to the commonly used homeostasis model assessment of insulin resistance (HOMA-IR) index [17]. Its predictive performance has been shown to be even better than that of HOMA-IR in a study that was also validated by a hyperglycemic clamp [18].

The TyG index has been shown to increase the risk of CRC [21–24], and another study has shown that the TyG index can be used to predict the risk of colorectal neoplasms in patients without CVD [25]. However, few studies have investigated the association between IR-related indicators and recurrent CRA incidence, and the results are inconsistent [26–28]; moreover, the association of the TyG index, a surrogate marker of IR, with recurrent CRA has been unclear. Based on these findings, we hypothesized that an increased TyG index would contribute to CRA development, such as an increased risk of recurrent adenomas. We therefore assessed the association between the TyG index and the occurrence and recurrence of CRA in this study.

## Methods

### Study population

**Fig. 1:**
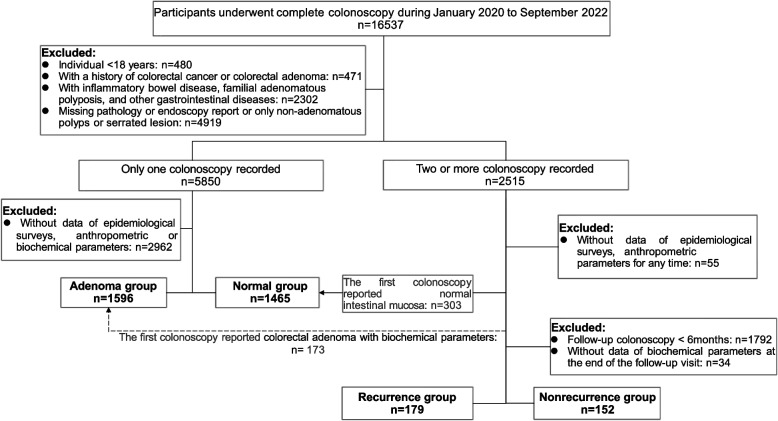
Flow chart of the study population

We retrospectively analyzed 16537 participants who underwent complete colonoscopy at a hospital in China from January 2020 to September 2022. Patients were excluded if they were younger than 18 years of age, had incomplete basic information or lacked biochemical parameters, did not have pathology or endoscopy reports, or were diagnosed only with nonadenomatous polyps or serrated polyps. In addition, subjects with a previous history of CRC or CRA, concomitant inflammatory bowel disease, familial adenomatous polyposis, or other gastrointestinal diseases were also excluded. A total of 3392 participants in four groups were enrolled in the study (Fig. 1).

Participants’ basic information, biochemical indices and adenoma characteristics were collected through questionnaires or electronic medical records and included sex, age, height, weight, body mass index (BMI), systolic blood pressure (SBP), diastolic blood pressure (DBP), clinical symptoms (change in stool habits or characteristics), lifestyle habits (history of smoking and drinking), family history, personal disease history and biochemical indicators such as FBG, total cholesterol (TC), TG, high-density lipoprotein cholesterol (HDL-C), low-density lipoprotein cholesterol (LDL-C), and number, size, location, histology and progression of adenomas. The TyG index was calculated as LN [TG (mg/dl) × FBG (mg/dl)/2] [16].

### Covariates

A BMI ≥ 28 kg/m2 was classified as obesity [29]; blood pressure ≥ 140/90 mmHg or self-reported previously diagnosed hypertension or use of antihypertensive medication as hypertension [30]; FBG ≥ 6.1 mmol/L or self-reported previously diagnosed diabetes or use of antidiabetic agents as hyperglycemia [31]; TC ≥ 5.2 mmol/L; TG ≥ 1.7 mmol/L; HDL-C < 1.0 mmol/L; LDL-C ≥ 3.4 mmol/L for any of these or self-reported previous diagnoses of hyperlipidemia or current use of lipid-lowering agents as dyslipidemia [32]; an average of > 10 cigarettes and > 40g of alcohol per day for five consecutive years was considered to be current smoking or drinking habits.

### Adenoma characteristics and outcomes

Colonoscopy was considered adequate based on the report of adequate bowel preparation and completion of cecal intubation. The histological classification of adenomas was based on the fifth edition of the World Health Organization Classification of Tumors of the Digestive System [33]. The histology, number, size, and location of all adenomas were determined from the endoscopy report. Adenomas were classified as proximal colon (from the cecum to and including the splenic flexure), distal colon or rectum (from the splenic flexure and including the rectum), or both (more than 1 adenoma located at both sites). Adenoma histology revealed tubular, tubulovillous or villous adenomas according to their villus content, with the highest degree of villi being recorded for more than 1 adenoma. The number and size of pathologically confirmed endoscopically reported adenomas were recorded, and the largest diameter was used for more than 1 adenoma. Adenoma heterogeneity was divided into high-grade and low-grade dysplasia. We classified the higher-risk categories of adenomas into two categories [34]: advanced adenoma (adenomas ≥ 10 mm in size, tubulovillous or villous histology, or high-grade dysplasia) and 2 or more adenomas. We considered a first diagnosis and resection of CRA, followed by redetection of the adenoma in situ or ectopically on colonoscopy at least six months apart as recurrent adenoma and normal intestinal mucosa on repeat examination as nonrecurrence of adenoma.

### Statistical analyses

We compared the characteristics of recurrent adenoma patients with nonrecurrent adenoma and CRA patients with normal intestinal mucosa stratified by TyG quartiles (continuous variables: one-way analysis of variance or nonparametric test; categorical variables: chi-square test). Multivariate logistic regression models were used to estimate odds ratios (ORs) and 95% confidence intervals (CIs) for the association between TyG index and CRA occurrence and recurrence risk. Model^a^ was adjusted for basic subject information such as sex, age and BMI; model^b^ was further adjusted for disease history, family history, lifestyle and biochemical indices; and model^c^ was further adjusted for the characteristics of the removed adenoma on the basis of the previous ones when studying recurrence. The selection of adjustment variables was informed by clinical knowledge and previous literature and followed the results of univariate logistic regression. Additionally, ORs and 95% CIs were also estimated for subjects with recurrent adenoma in different locations or higher risk categories and for those in the nonrecurrence group. We tested the linear trend by entering the median value of each quartile of the TyG index as a continuous variable in the models. A restricted cubic spline (RCS) was applied to fit the dose‒response relationship between CRA recurrence and the TyG index.

We conducted subgroup analyses based on sex, age, BMI and comorbidities to assess the association of the TyG index with recurrent adenomas in specific populations and analyzed interactions. In addition, due to the limited sample size, we investigated the association between age and the risk of adenoma recurrence by dividing participants at intervals greater than a certain value by 5 years. ORs and 95% CIs for TyG index components (FBG and TG) were also estimated to provide additional information for the risk assessment of adenoma recurrence. The discriminatory power of the TyG index for predicting later recurrence was assessed with the area under the receiver operating characteristic (ROC) curve using 170 patients with a TyG index at initial adenoma diagnosis. In addition, we excluded participants with extreme TyG index values (those whose measurements deviated from the mean by more than 3 times the standard deviation) or those with less than one year of follow-up from sensitivity analyses to ensure robust results. Analyses were performed using R 4.2.1 and SPSS v25.0. A two-tailed test was used, and *P* < 0.05 was considered to indicate statistical significance.

## Results

### Characteristics of the participants

The study included 3392 participants, 1596 of whom had CRA, 1465 had normal intestinal mucosa, 179 had recurrent adenomas and 152 had nonrecurrent adenomas. The characteristics of the participants in the recurrence analysis are presented in Table 1. The number of people diagnosed with recurrent adenoma increased; BMI, FBG, TC, TG, and LDL-C increased; and HDL-C decreased according to TyG quartiles. Compared to participants in lower quartiles, those in higher quartiles were more commonly male, alcohol drinkers, obese, hyperglycemic or dysplastic. The difference in the characteristics of resected adenomas between the quartiles of the TyG index was not statistically significant. Subject characteristics in the occurrence analysis were similar, as detailed in Table S1. The characteristics of the participants in the two groups are shown in Table S2. The adenoma and recurrence groups were more likely to be male; be older; be smokers; have hypertension or dyslipidemia; have higher FBG, TG and TyG levels; and have lower HDL-C levels than were the control groups.

**Table 1:** Characteristics stratified by TyG quartiles among participants included in the recurrence analysis

Variable	Q1(*n* = 83)	Q2(*n* = 82)	Q3(*n* = 81)	Q4(*n* = 85)	*P* value
TyG index, range	< 8.28	8.28–8.62	8.63–8.92	> 8.92	
Sex					0.001
Female, n (%)	35 (42.17)	29 (35.37)	20 (24.69)	13 (15.29)	
Male, n (%)	48 (57.83)	53 (64.63)	61 (75.31)	72 (84.71)	0.001
Age, y, mean (SD)	56.54 (10.61)	54.77 (10.82)	53.63 (10.30)	55.11 (9.65)	0.348
Change in stool habits or characteristics, n (%)	21 (25.30)	10 (12.20)	17 (20.99)	19 (22.35)	0.182
Cancer history, n (%)	12 (14.46)	2 (2.44)	3 (3.70)	8 (9.41)	0.012
Obesity, n (%)	5 (6.02)	11 (13.41)	17 (20.99)	28 (32.94)	< 0.001
Hyperglycemia, n (%)	7 (8.43)	12 (14.63)	15 (18.52)	37 (43.53)	< 0.001
Hypertension, n (%)	25 (30.12)	33 (40.24)	29 (35.80)	41 (48.24)	0.102
Dyslipidemia, n (%)	24 (28.92)	30 (36.59)	48 (59.26)	83 (97.65)	< 0.001
Current smoking, n (%)	10 (12.05)	15 (18.29)	12 (14.81)	17 (20.00)	0.509
Current drinking, n (%)	15 (18.07)	13 (15.85)	18 (22.22)	30 (35.29)	0.013
Family history of CRC, n (%)	3 (3.61)	6 (7.32)	1 (1.23)	1 (1.18)	0.093
Family history of cancer, n (%)	11 (13.25)	11 (13.41)	8 (9.88)	10 (11.76)	0.891
SBP, mmHg, mean (SD)	124.22 (14.53)	125.85 (13.17)	127.01 (13.64)	126.53 (13.68)	0.584
DBP, mmHg, mean (SD)	76.25 (10.48)	77.59 (11.21)	79.23 (9.97)	79.62 (10.34)	0.142
BMI, kg/m^2^, mean (SD)	23.8 (2.70)	25.08 (3.43)	25.99 (2.72)	26.67 (3.07)	< 0.001
FBG, mmol/L, mean (SD)	4.62 (0.65)	4.97 (0.67)	5.40 (0.98)	6.36 (2.03)	< 0.001
TC, mmol/L, mean (SD)	4.07 (0.91)	4.30 (0.88)	4.61 (0.84)	4.67 (0.92)	< 0.001
TG, mmol/L, median (Q1, Q3)	0.86 (0.72,1.00)	1.18 (1.08,1.35)	1.55 (1.43,1.66)	2.26 (1.85,3.38)	< 0.001
HDL-C, mmol/L, mean (SD)	1.36 (0.35)	1.24 (0.27)	1.12 (0.24)	1.00 (0.21)	< 0.001
LDL-C, mmol/L, mean (SD)	2.38 (0.77)	2.58 (0.71)	2.91 (0.75)	2.73 (0.80)	< 0.001
Characteristics of the removed adenoma					
Location, n (%)					0.636
Distal colon or rectum	40 (48.19)	36 (43.90)	39 (48.15)	34 (40.00)	
Proximal colon	37 (44.58)	39 (47.56)	33 (40.74)	38 (44.71)	
Both^a^	6 (7.23)	7 (8.54)	9 (11.11)	13 (15.29)	
Histology, n (%)					0.86
Tubular	66 (79.52)	68 (82.93)	63 (77.78)	67 (78.82)	
Tubulovillous	17 (20.48)	14 (17.07)	18 (22.22)	18 (21.18)	
No. of adenomas, n (%)					0.211
1	72 (86.75)	69 (84.15)	68 (83.95)	62 (72.94)	
2	10 (12.05)	12 (14.63)	10 (12.35)	18 (21.18)	
≥ 3	1 (1.20)	1 (1.22)	3 (3.70)	5 (5.88)	
Size, n (%)					0.21
1–5 mm	40 (48.19)	39 (47.56)	34 (41.98)	30 (35.29)	
6–9 mm	17 (20.48)	28 (34.15)	22 (27.16)	29 (34.12)	
≥ 10 mm	23 (27.71)	11 (13.41)	21 (25.93)	19 (22.35)	
Unspecified	3 (3.61)	4 (4.88)	4 (4.94)	7 (8.24)	
Highgrade, n (%)	5 (6.02)	6 (7.32)	11 (13.58)	3 (3.53)	0.091
Advanced adenoma, n (%)	32 (38.55)	21 (25.61)	26 (32.10)	27 (31.76)	0.365
Followup, d, median (Q1, Q3)	384 (331,520)	413 (353,557)	405 (303,510)	415 (333,619)	0.277
Recurrent adenoma, n (%)	33 (39.76)	35 (42.68)	49 (60.49)	62 (72.94)	< 0.001

*SBP* systolic blood pressure, *DBP* diastolic blood pressure, *BMI* body mass index, *FBG* fasting blood glucose, *TC* total cholesterol, *TG* triglyceride, *HDL-C* high-density lipoprotein cholesterol, *LDL-C* low-density lipoprotein cholesterol, *TyG* triglyceride-glucose, *CRC* colorectal cancer

^a^More than 1 adenoma was located in both the distal colon or rectum and proximal colon

### Associations between the TyG index and risk of CRA

Univariate and multivariate logistic regression analyses revealed that sex, age, hypertension, and the TyG index were independent risk factors for CRA occurrence (Table S3). Multivariate-adjusted logistic regression was used to further explore the independent effect of the TyG index on CRA occurrence (Table 2). A one-unit increase in the TyG index increased the risk of CRA by 22.5% (95% CI 1.027–1.460). As a categorical variable, there was a greater risk of adenoma in the second (Q2), third (Q3) and highest quartile (Q4) of the TyG index than in the lowest quartile (Q1), with ORs of 1.324 (95% CI 1.020–1.718), 1.349 (95% CI 1.030–1.765), and 1.445 (95% CI 1.055–1.980), respectively, and a linear trend (*p* = 0.026). In addition, the TyG index was more strongly correlated with the occurrence of advanced adenomas. A one-unit increase in the TyG index increased the risk of advanced adenoma by 24.5% (95% CI 1.013–1.529). The risk of advanced adenomas in the participants in Q4 of TyG was 1.481 (95% CI 1.022–2.146) times greater than that in the Q1 group. The results were adjusted for potential confounding factors.

**Table 2:** ORs and 95%CIs for adenoma occurrence according to the TyG index

	Case/total	Crude		Model^a^		Model^b^	
		OR(95%CI)	*P* value	OR(95%CI)	*P* value	OR(95%CI)	*P* value
Any adenoma							
TyG index (continuous)	1596/3061	1.268(1.136–1.415)	< 0.001	1.395(1.212–1.607)	< 0.001	1.225(1.027–1.460)	0.024
Quartiles of TyG index							
Q1(< 8.13)	346/774	1.000(reference)		1.000(reference)		1.000(reference)	
Q2(8.13–8.50)	410/761	1.445(1.182–1.767)	< 0.001	1.380(1.067–1.787)	0.014	1.324(1.020–1.718)	0.035
Q3(8.51–8.94)	413/754	1.498(1.225–1.833)	< 0.001	1.489(1.153–1.923)	0.002	1.349(1.030–1.765)	0.03
Q4(> 8.94)	427/772	1.531(1.253–1.871)	< 0.001	1.818(1.402–2.358)	< 0.001	1.445(1.055–1.980)	0.022
p for trend^c^			< 0.001		< 0.001		0.026
Advanced adenoma							
TyG index (continuous)	690/2155	1.332(1.161–1.528)	< 0.001	1.458(1.235–1.721)	< 0.001	1.245(1.013–1.529)	0.037
Quartiles of TyG index							
Q1(< 8.13)	144/572	1.000(reference)		1.000(reference)		1.000(reference)	
Q2(8.13–8.50)	161/512	1.363(1.045–1.778)	0.022	1.299(0.952–1.772)	0.099	1.238(0.905–1.695)	0.182
Q3(8.51–8.94)	193/534	1.682(1.299–2.178)	< 0.001	1.664(1.228–2.254)	0.001	1.483(1.078–2.040)	0.016
Q4(> 8.94)	192/537	1.654(1.277–2.142)	< 0.001	1.938(1.423–2.640)	< 0.001	1.481(1.022–2.146)	0.038
p for trend^c^			< 0.001		< 0.001		0.026

*TyG* triglyceride-glucose

^a^Adjusted for sex, age, and systolic blood pressure

^b^ Further adjusted for history of cancer, hypertension, hyperglycemia, dyslipidemia, history of smoking, family history of colorectal cancer, and high-density lipoprotein cholesterol

^c^ Tested for linear trend by entering the median value of each quartile of the TyG index as a continuous variable in the models

### Associations between the TyG index and risk of CRA recurrence

**Fig. 2:**
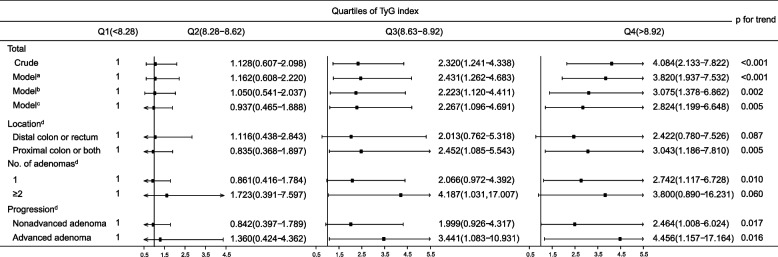
The TyG index is associated with the risk of adenoma recurrence. **a** Adjusted for sex and age. **b** Further adjusted for obesity, hypertension, dyslipidemia, current smoking and drinking status, and high-density lipoprotein cholesterol. **c** Further adjusted for location, number, histology, size, and progression of the removed adenomas; 18 participants with missing data on the size of the removed adenomas were excluded. **d** Multinomial logistic regression based on multivariate adjustment

Stepwise selection analysis revealed that sex, age, excised adenoma size, and TyG index were found to be independent risk factors for recurrent adenoma (Table S4). The associations of the TyG index with the risk of adenoma recurrence are shown in Fig. 2. Participants with Q3 (OR 2.267, 95% CI 1.096–4.691) and Q4 (OR 2.824, 95% CI 1.199–6.648) of the TyG index had a greater risk of adenoma recurrence than did those with Q1, and there was a linear trend (*p* = 0.005) adjusted for potential confounding factors. Compared to subjects without recurrent adenomas, patients with recurrent adenomas had a greater risk of recurrence in higher-risk categories (advanced adenoma, 2 or more adenomas), the proximal colon or both adenomas according to the increasing TyG quartiles. However, at Q3, when the TyG index increased the risk of recurrence of 2 or more adenomas (OR 4.187, 95% CI 1.031–17.007), Q4 had no significant effect. However, when TyG index extremes were removed, both Q3 (OR 4.385, 95% CI 1.055–18.231) and Q4 (OR 5.079, 95% CI 1.136–22.714) significantly increased the risk of 2 or more recurrent adenomas (Table S5).

We also investigated the correlation between recurrent adenoma and TyG index components, including FBG and TG levels (Table S6). After adjusting for potential confounders, the association between FBG and adenoma recurrence was not significant, and the high TG group was associated with an increased risk of recurrence.

**Fig. 3:**
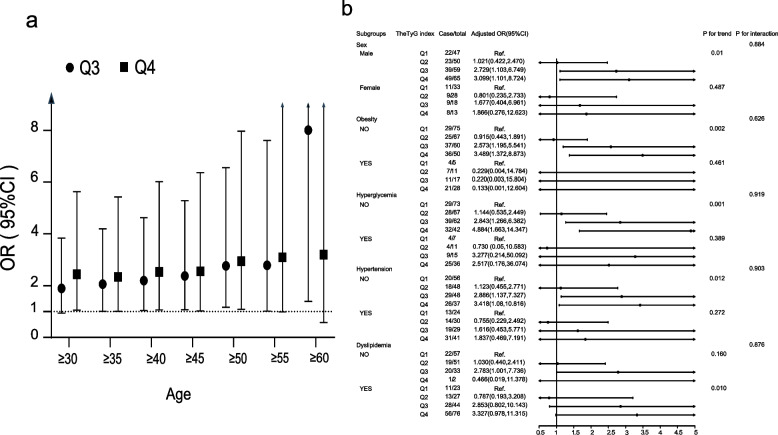
Subgroup analysis of the association of the TyG index with adenoma recurrence. **a** Stratified by age (older than a certain value according to a 5-year interval). **b** Sexually stratified, obesity, hyperglycemia, hypertension, dyslipidemia. All the analyses were adjusted for sex, age, obesity, hypertension, dyslipidemia, current smoking and drinking status, high-density lipoprotein cholesterol, location, number, histology, size, and progression of removed adenomas

We also conducted subgroup analyses on specific populations. Multivariate analysis revealed that the TyG index was associated with an increased risk of adenoma recurrence throughout the age range, with ORs increasing with age (Fig. 3a). However, there was an association between the TyG index and an increased risk of adenoma recurrence in male participants, or without obesity, hyperglycemia, hypertension or dyslipidemia (Fig. 3b). There was no interaction between the TyG index and any of these variables.

**Fig. 4:**
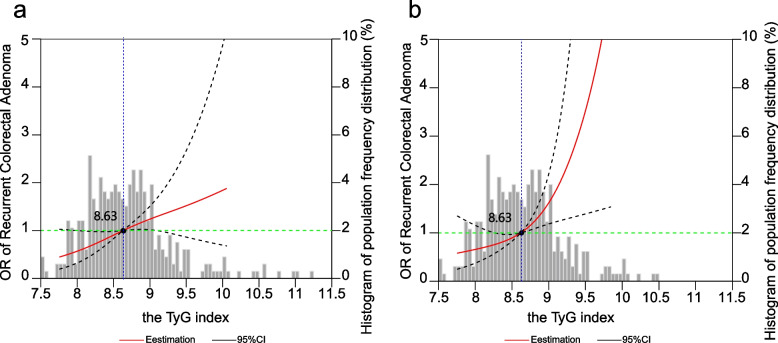
Restricted cubic spline between recurrent adenomas and the TyG index. **a** Using all the data; **b** 6 participants with extreme TyG index values were excluded. All the analyses were adjusted for sex, age, obesity, hypertension, dyslipidemia, current smoking and drinking status, high-density lipoprotein cholesterol, location, number, histology, size, and progression of removed adenomas

Figure 4 illustrates the association between the TyG index and recurrent adenoma incidence. There was an increased risk of CRA recurrence for patients with a TyG index above 8.63, indicating a linear association (*p*-nonlinear > 0.05). However, as the TyG index increased, the 95% CI showed a trend toward 1 (Fig. 4a), which was significant when we excluded the extreme values of the TyG index (Fig. 4b).

**Fig. 5:**
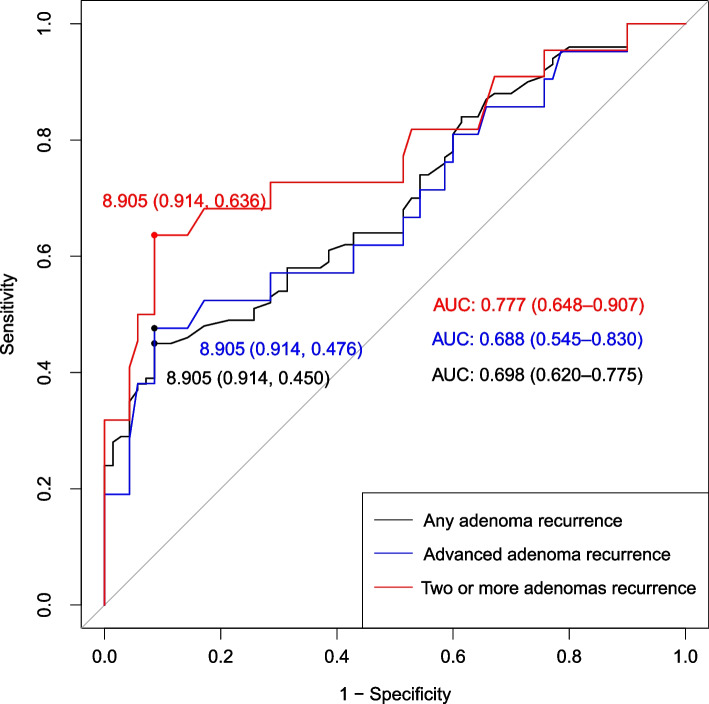
ROC curve for the use of the TyG index for the diagnosis of recurrent adenoma. A total of 170 subjects had TyG at the initial diagnosis of adenoma. The data from 170 patients were used to diagnose the recurrence of any adenoma, that from 91 patients was used to diagnose the recurrence of advanced adenomas, and that from 92 patients was used to diagnose the recurrence of two or more adenomas

The ability of the TyG grade to predict adenoma recurrence was evaluated using ROC curves (Fig. 5). Compared to nonrecurrence, the TyG index had good predictive diagnostic efficacy for any adenoma recurrence (AUC 0.698, 95% CI 0.620–0.775), advanced adenoma recurrence (AUC 0.688, 95% CI 0.545–0.830), and two or more adenoma recurrences (AUC 0.777, 95% CI 0.648–0.907). According to the sensitivity analysis, a stronger correlation between the TyG index and the risk of adenoma recurrence was observed after excluding six participants with extreme TyG index values or 119 participants with less than one year of follow-up (Table S7).

## Discussion

The aim of this study was to investigate the association of the TyG index with CRA occurrence and recurrence. We found that a greater risk of CRA occurrence and recurrence was associated with a higher TyG index, and the robustness of the results was further validated by sensitivity studies, which are consistent with the theory that IR promotes CRC development.

However, few studies have investigated the role of the TyG index in this area. Josef Fritz et al. [24] analyzed 510,471 participants from six European cohorts and reported that the TyG index was associated with an increased risk of digestive system cancers, including CRC. Takuro Okamura et al. [21] reported that the TyG index could predict the onset of CRC in a retrospective cohort study of 27944 participants. Recently, several studies have explored the value of the TyG index in this area, but the relationship between this index and colorectal tumors has been revealed [22, 23, 25]. Our study reports similar results for an increased risk of CRA with an elevated TyG index.

Our study did not investigate the underlying mechanism, but it may be related to IR. The insulin and insulin-like growth factor axis promotes tumor progression through direct proproliferative effects and indirectly through alterations in glucose metabolism [11]. Insulin may also promote tumor formation by upregulating acyl-coenzyme A: cholesterol acyltransferase-1, which mediates cell proliferation and metastatic effects on CRC cells [10], increasing vascular cell adhesion molecule-1 expression in tumor endothelial cells, which changes the homing of other immune cells to the tumor microenvironment [9].

Little attention has been given to the relationship between insulin-related indicators and adenoma recurrence, and the available literature is controversial. ANDREW FLOOD et al. [27] reported a greater risk of adenoma recurrence in patients with elevated serum insulin and glucose and an even greater risk of advanced adenoma recurrence in those with elevated glucose; however, some of these findings do not support a role for insulin biomarkers or recurrent CRA [26, 28]. Our study showed that subjects in the TyG Q3 and Q4 cohorts had a significantly greater risk of adenoma recurrence than did those in the Q1 cohort. RCS analysis also showed that the TyG index increased the risk of adenoma recurrence from Q3 onwards. For the higher-risk categories, the recurrence risk of advanced adenoma or 2 or more adenomas was approximately 4–5 times greater than that for the lower-risk categories, and the risk of proximal or both recurrences was similarly greater than that for distal or rectal adenomas, with no significant difference between distal or rectal adenoma recurrence and nonrecurrence. These results were adjusted for multiple confounding factors, including the characteristics of the removed adenomas. Many studies have used 3 or more adenomas as a high-risk category [34]. The diagnosis of adenomas in this study was based on pathological findings; however, not all polyps were pathologically examined; therefore, very few participants had 3 or more adenomas at the same time, so 2 or more were used as one of the higher-risk categories in this study.

In our study, the TyG index was found to significantly increase the risk of adenoma recurrence in males compared with females, possibly because of differences in sex hormones. Estrogen has potential preventive and therapeutic effects on sporadic CRC and familial adenomatous polyposis [35]. Another recent study indicated a potential role for sex hormones in the early stages of colorectal carcinogenesis [36]. We found that the TyG index increased the risk of adenoma recurrence throughout the age range, suggesting that follow-up of the TyG index after initial resection of adenomas was beneficial to the individual regardless of age, although the risk of adenoma recurrence increased with age as the TyG index increased. Obesity, hyperglycemia, hypertension, and dyslipidemia may increase the risk of recurrence, which is consistent with the available evidence [27, 37–39]. However, interestingly, when we restricted the recurrence analysis to certain populations, the TyG index was observed to increase the risk of adenoma recurrence only in participants without obesity, hyperglycemia, hypertension or dyslipidemia. The presence of underlying disease may attenuate the association between the TyG index and CRA recurrence. However, similar results were found in a prospective cohort study investigating the association between the TyG index and CRC [23], which may suggest that the TyG index may play a more prominent role in relatively healthy populations, coinciding with the original findings of the indicator [16], a finding that provides a new perspective on early screening for CRC.

The main strength of the study is that it provides a unique perspective on the association between IR and CRA recurrence and even future CRC screening. The TyG index is a relatively low-cost parameter that measures FBG and TG in all clinical laboratories and does not require the quantification of serum insulin levels (an expensive test), making it a promising alternative indicator of IR in mass screening due to its easy availability. In addition, sensitivity and subgroup analyses were performed to ensure the robustness of the results. This study has several limitations. First, the sample size was small, and the data were collected from a single hospital; therefore, the results should be further validated in large samples. Second, the number of adenomas in our study was based on pathological findings, but not all adenomas were actually examined; therefore, the results may be underestimated. In addition, serrated polyps were not included in this study, although they have recently received much attention because the diagnosis of serrated polyps is extremely rare, probably because of their more insidious morphology, which makes them easy to miss. Third, because of the retrospective nature of the study, we were unable to confirm the sequence of the TyG index or end points or collect the incidence and recurrence rates of adenomas. However, most of the data in this study were collected from electronic medical records without recall bias, and the ability of the baseline TyG score to predict subsequent adenoma recurrence was assessed in 170 patients. We will continue to explore its predictive value for adenoma recurrence, and its value is expected to be validated in large cohort studies in the future. Fourth, this study did not take into account all influencing factors, such as the inability to collect drugs that affect metabolism, which may have affected the final results. Fifth, we did not follow up long enough and may not be able to observe all recurrences. However, a recent study showed no statistically significant difference in recurrence between individuals who were monitored at 1 vs. 3 years for advanced colorectal neoplasia [40]. Finally, adenomas missed on baseline colonoscopy may be considered recurrent in some cases; however, studies have shown that large polyps are missed at a much lower rate than small polyps [41], and in normal daily practice, only a small number of clinically important adenomas are missed [42]. Our findings support a stronger correlation between the TyG index and advanced adenoma.

## Conclusion

Our results indicate that an increase in the TyG index is associated with an increased risk of adenoma occurrence and recurrence, with a stronger association with the latter. It may be beneficial to monitor the TyG index in patients with a previous diagnosis of adenoma, especially in men or without underlying disease.

### Supplementary Information

**Supplementary Material 1. **

**Supplementary Material 2. **

**Supplementary Material 3. **

**Supplementary Material 4.**

**Supplementary Material 5. **

**Supplementary Material 6. **

**Supplementary Material 7. **

## Data Availability

The datasets generated or analyzed during the current study are not publicly available but are available from the corresponding author upon reasonable request.

## Abbreviations

CRC: Colorectal cancer
CRA: Colorectal adenoma
TyG: Triglyceride-glucose
IR: Insulin resistance
HOMA-IR: Homeostasis model assessment of insulin resistance
RCS: Restricted cubic spline
BMI: Body mass index
SBP: Systolic blood pressure
DBP: Diastolic blood pressure
FBG: Fasting blood glucose
TC: Total cholesterol
TG: Triglycerides
HDL-C: High-density lipoprotein cholesterol
LDL-C: Low-density lipoprotein cholesterol
ORs: Odds ratios
CIs: Confidence intervals
